# CircRNA expression pattern and circRNA-miRNA-mRNA network in the pathogenesis of nonalcoholic steatohepatitis

**DOI:** 10.18632/oncotarget.12186

**Published:** 2016-09-22

**Authors:** Xi Jin, Chun-yan Feng, Zun Xiang, Yi-peng Chen, You-ming Li

**Affiliations:** ^1^ Department of Gastroenterology, The First Affiliated Hospital, School of Medicine, Zhejiang University, Hangzhou, Zhejiang, China; ^2^ Department of Clinical Medicine, Hangzhou Medical College, Hangzhou, China

**Keywords:** NASH, microarray, qRT-PCR, circRNA, miRNA, Pathology Section

## Abstract

The pathogenesis of nonalcoholic steatohepatitis (NASH) is still unclear, where involvement of circRNA is considered for its active role as “miRNA sponge”. Therefore, we aimed to investigate the circRNA expression pattern in NASH and further construct the circRNA-miRNA-mRNA network for in-depth mechanism exploration. Briefly, NASH mice model was established by Methionine and choline deficiency (MCD) diet feeding. Liver circRNA and mRNA profile was initially screened by microarray and ensuing qRT-PCR verification was carried out. The overlapped predicted miRNAs as downstream targets of circRNAs and upstream regulators of mRNAs were verified by qRT-PCR and final circRNA-miRNA-mRNA network was constructed. Gene Ontology (GO) and KEGG pathway analysis were further applied to enrich the huge mRNA microarray data. To sum up, there were 69 up and 63 down regulated circRNAs as well as 2760 up and 2465 down regulated mRNAs in NASH group, comparing with control group. Randomly selected 13 of 14 mRNAs and 2 of 8 circRNAs were successfully verified by qRT-PCR. Through predicted overlapped miRNA verification, four circRNA-miRNA-mRNA pathways were constructed, including circRNA_002581-miR-122-Slc1a5, circRNA_002581- miR-122-Plp2, circRNA_002581-miR-122-Cpeb1 and circRNA_007585-miR-326- UCP2. GO and KEGG pathway analysis also enriched specific mRNAs. Therefore, circRNA profile may serve as candidate for NASH diagnosis and circRNA-miRNA -mRNA pathway may provide novel mechanism for NASH.

## INTRODUCTION

Nonalcoholic steatohepatitis (NASH) is considered as an important stage of nonalcoholic fatty liver disease (NAFLD) that is characterized by lipid deposition in hepatocytes of liver parenchyma without alcohol over-consumption and composed of simple steatosis, NASH, fibrosis and cirrhosis with disease progression. Increasing evidences suggest that NASH acts as the “watershed” in NAFLD progress, where the percentage of progressing from NASH into liver fibrosis and end-stage liver diseases have greatly increased into 41% and 5.4%, respectively [1]. Furthermore, NASH is also regarded as the major cause of cryptogenic cirrhosis. Therefore, it is of clinical and theoretical importance for in-depth NASH study.

Currently, though the “two hit” hypothesis has outlined the mainstream pathogenesis of NAFLD [2], the detailed mechanism of NASH is still vague and become hot research spot [3]. With the development of high throughput technology such as microarray, we [4] and others [5, 6] identified the miRNA profile of NASH, supporting the importance of epigenomic regulation in NASH pathogenesis and progress. Compared with miRNA, circular RNA (circRNA) is one type of rediscovered endogenous noncoding RNA and become a hotspot in RNA research. Unlike linear RNA that is terminated with 5′ cap and 3′ tale, circRNA forms covalently closed continuous loop structure through specific splicing method and is considered as the major subtype in gene transcription[7]. Increasing evidences suggest that the characteristics of circRNA include: existing in nearly all types of species; expressing in a tissue and disease dependent manner; more stable in tissue and circulation than linear RNA by resisting RNAase degradation [8, 9]. All these traits make circRNA expression profile become a good candidate as molecular diagnostic biomarker for various diseases such as gastric, ovarian and colon cancers [10, 11].

Another peculiarity of circRNA is its “miRNA sponge” function, where it can efficiently bind and inhibit miRNA transcription, further influence downstream mRNA expression and finally participate in various diseases [12]. Previous study showed that a specific circRNA-cANRIL was the independent risk factor for atherosclerosis [13] that was regarded as the cardiovascular phenotype of metabolic syndrome (MS). Nevertheless, though considered as the hepatic manifestation of MS, neither the circRNA expression pattern nor the specific circRNA function of NAFLD has been reported. Therefore, considering the importance of NASH in NAFLD, we investigated the circRNA profile and constructed several circRNA-miRNA-mRNA pathways in a well developed NASH mice model through combination of microarray screening, qRT- PCR verification and bioinformatics, aiming to provide novel data for NASH diagnosis and pathogenesis.

## RESULTS

### Successful establishment of NASH mice model

**Figure 1:**
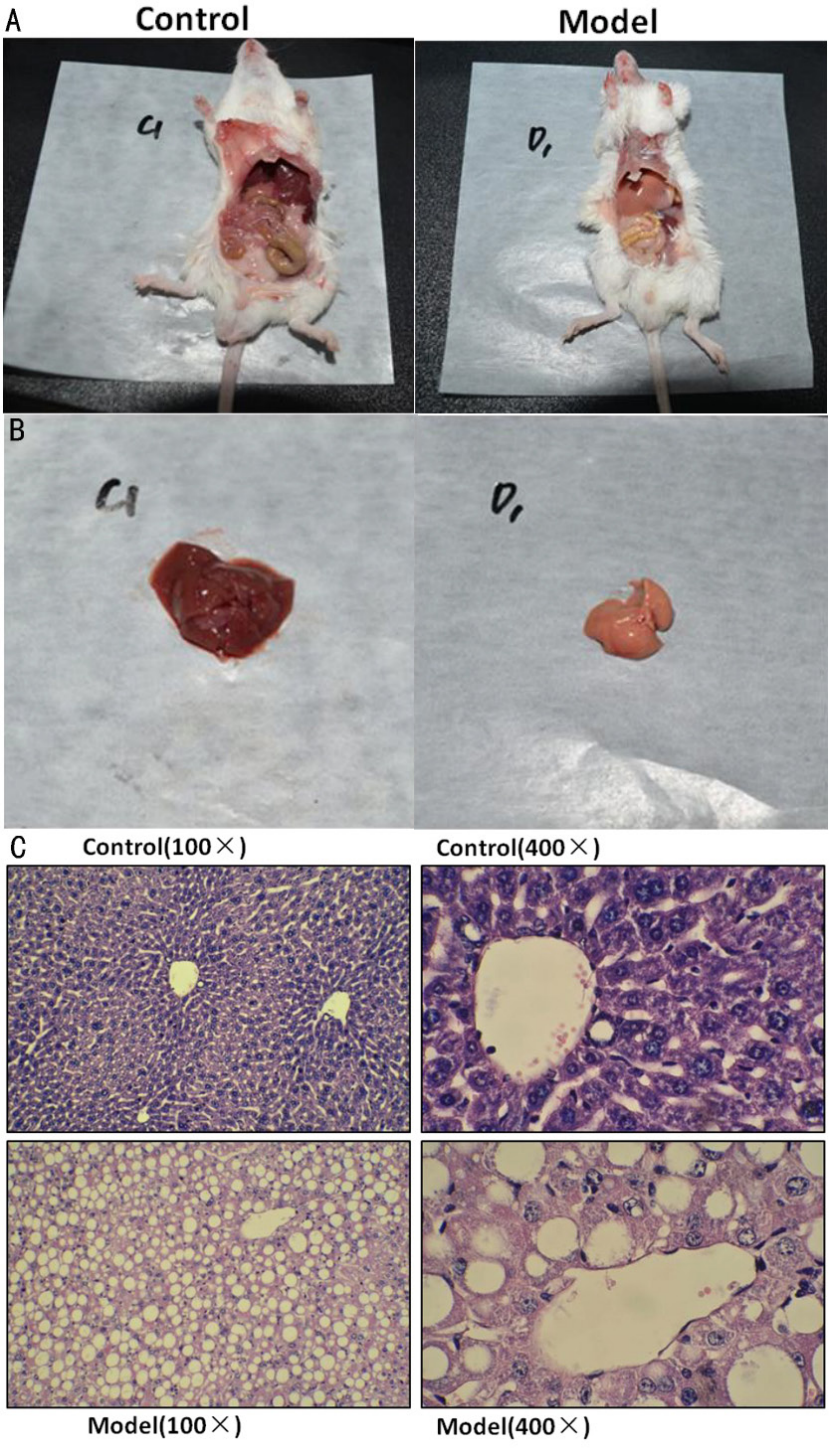
successfully established NASH mice model Representative images of mice body **A.**, liver tissue **B.** and H-E staining with different magnification **C.**

NASH mice model was successfully established after feeding MCD diet for 4 weeks, as reflected by distinctive changes in serum biomarker and liver tissue. Generally, mice from NASH group showed yellow-enlarged liver as well as hepatic fat deposition, hepatocyte ballooning, mild to moderate chronic portal and intra-acinar inflammation (Figure 1), as confirmed by an independent pathologist. Besides, though body weight and liver weight were both significantly decreased in NASH group for malnutrition, HAI was still significantly increased. Finally, serum ALT and AST levels were significantly increased while TG was decreased in NASH group (Table 1).

**Table 1:** Changes in hepatic and serologic markers in NASH animal model

	Control	NASH	*p*
body weight (g)	24.17±1.11	12.55±0.39	<0.01
liver weight (g)	1.33±0.16	0.93±0.13	<0.01
HAI	0.055±0.007	0.074±0.01	<0.01
TG(mmol/L)	1.72±0.19	0.87±0.13	<0.01
ALT(mmol/L)	43.67±3.85	91.32±9.31	<0.01
AST(mmol/L)	51.03±5.18	103.44±11.25	<0.01

### Expression pattern of circRNA and mRNA in NASH mice model

**Figure 2:**
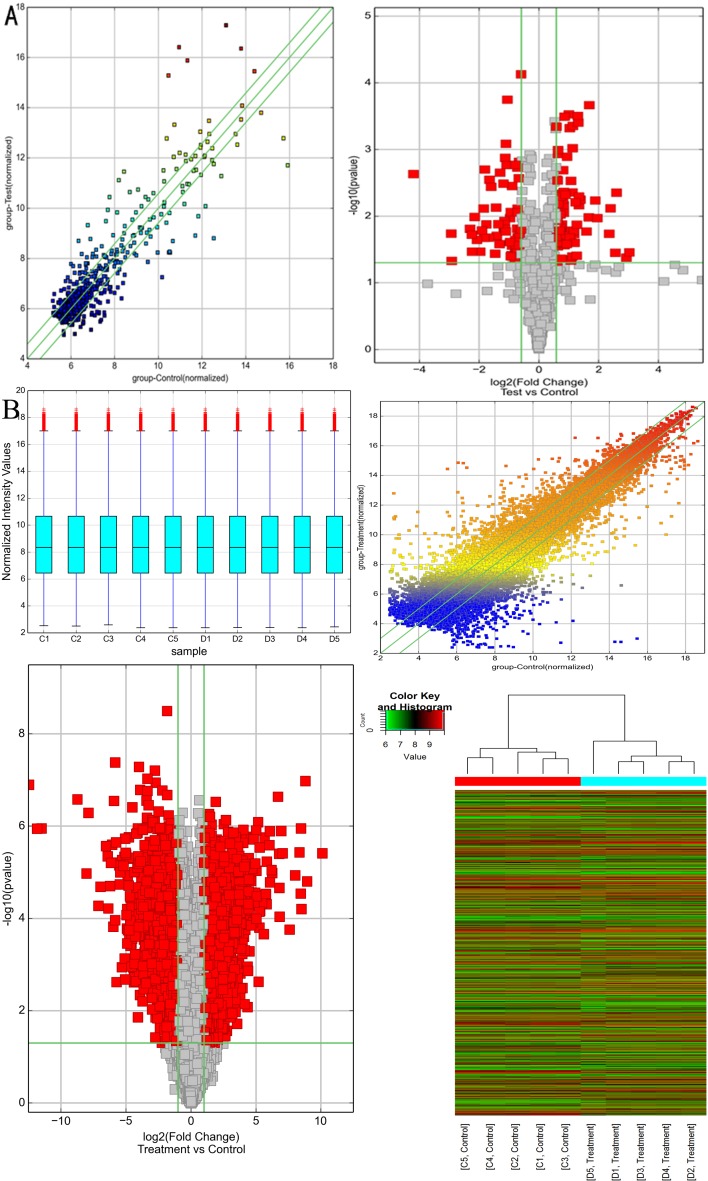
Bioinformatics analysis of differentially expressed circRNAs and mRNAs in NASH mice model **A.** Scatter plot (left panel) and volcano plot (right panel) showed the distributions of circRNAs in more direct way. **B.** Box plot (left upper panel), scatter plot (right upper panel), volcano plot (left lower panel) and heat map (right lower panel) showed the distributions of mRNAs in more direct way. After normalization, the distributions of log_2_ ratios among samples were nearly the same. The values of the X- and Y-axes in the scatter plot were the averaged normalized signal values of the group (log_2_ scaled). The green lines in scatter plot and volcano plot represent the default significant fold change (2.0). Hierarchical cluster analysis (heat map) of microarray data was used to assess the significant expression of mRNAs when comparing NASH with control. Red and green denoted high and low expression, respectively. Each RNA was represented by a single row of colored boxes and each sample was represented by a single column.

CircRNA and mRNA microarray were carried out in randomly selected 5 samples from each group. There were 69 up and 63 down regulated circRNAs (Supplementary Table S2) passing scatter and volcano plots (Figure 2A), where the top 10 circRNAs were summarized in Table 2 based on fold change. As jointly evaluated by box plot, scatter plot, volcano plot and heat map, the differential expression of mRNAs was also well categorized into NASH and control group (Figure 2B). Compared with the control group, there were 2760 up and 2465 down regulated mRNAs (fold change ≥2.0, P<0.05, Supplementary Table S3). The top 10 dys-regulated mRNAs were summarized in Table 3.

**Table 2:** Top 10 significantly dysregulated circRNAs in NASH

Name	Type	Best related linear transcript (Gene symbol)	Fold Change	*P* value
**Up regulation**				
mm9_circ_013935	intronic	ENSMUST00000178353	8.141	0.035
mm9_circ_002319	exonic	NM_021531(Carm1)	7.603	0.042
mm9_circ_016983	intragenic	NR_002142(Rpph1)	6.038	0.040
mm9_circ_009295	exonic	NM_027430(Mpc2)	5.879	0.035
mm9_circ_017649	exonic	NM_001037136(Agap1)	5.098	0.018
**Down regulation**				
mm9_circ_011775	antisense	uc012ath.2(Rn45s)	18.427	0.002
mm9_circ_011174	intragenic	TCONS_00013579 (XLOC_010800)	7.662	0.018
mm9_circ_006982	exonic	NM_031186(Ndst3)	7.555	0.046
mm9_circ_010484	exonic	NM_023060(Eefsec)	4.949	0.015
mm9_circ_011235	exonic	NR_003368(Pvt1)	4.743	0.019

### Computational analysis of significantly dys-regulated circRNAs and mRNAs

The significantly dys-regulated circRNAs were further categorized into different subgroups according to their genomic positions and effects. There were 7 antisense, 25 exonic, 28 intragenic and 9 intronic circRNAs of increased circRNAs and 7 antisense, 37 exonic, 15 intragenic and 4 intronic circRNAs of decreased circRNAs. Besides, miR-6418-5p, miR-7050-5p, miR-7118-5p, miR-6999-5p and miR-6923-3p were the predicted downstream miRNAs of circ_013935 that had the highest increased fold of 8.141 while miR-320-3p, miR-7051-5p, miR-6971-5p, miR-7652-5p and miR-3066-5p were the predicted downstream miRNAs of circ_011775 that had the highest decreased fold of 18.427. The other downstream miRNAs of significantly changed circRNAs were also summarized in Supplementary Table S2.

**Figure 3:**
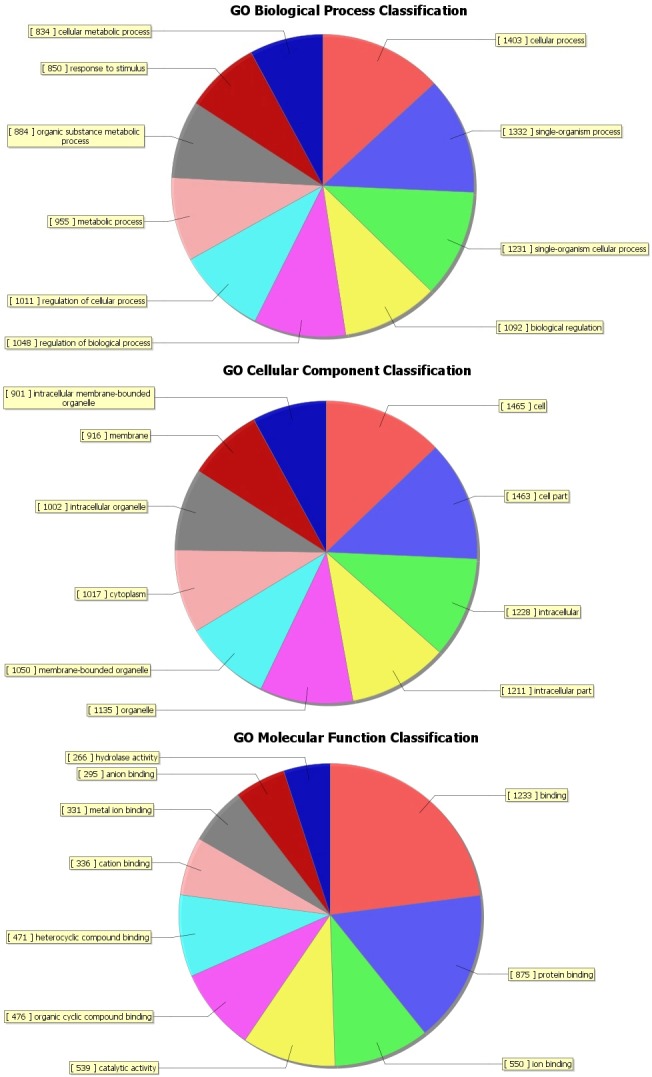
GO analysis of increased mRNAs with routine classification method The GO analysis categorized mRNAs into different groups under the theme of Biological process (BP), cellular component (CC) and molecular function (MF).

**Figure 4:**
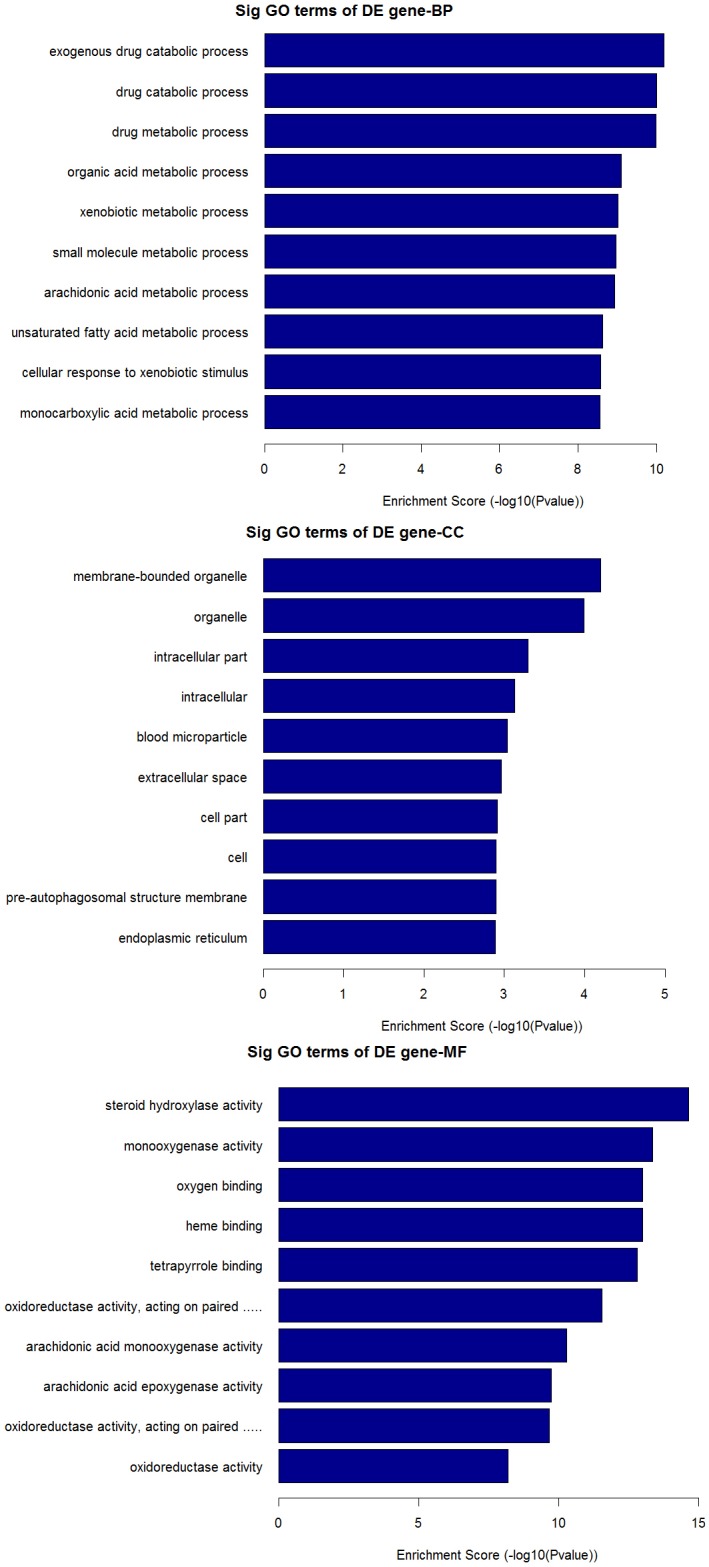
GO analysis of decreased mRNAs according to the values in the enrichment score under the theme of BP, CC and MF

**Figure 5:**
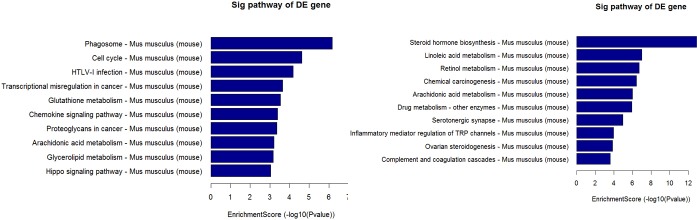
10 KEGG pathways of significantly up (left panel) and down (right panel) regulated mRNAs

The top 10 dys-regulated GO processes of each subgroup (BP, CC and MF) were analyzed according to enriched dys-regulated mRNAs. For increased mRNAs, the top 3 GO processes included cellular process, single-organism process and single- organism cellular process in BP; cell, cell part and intracellular in CC; binding, protein binding and ion binding in MF, according to the routine GO classification algorithms (Figure 3). Intriguingly, except for the replacement of protein binding by catalytic activity in MF, the other top 3 Go processes predicted by decreased mRNAs were the same as those predicted by increased mRNAs. Enrichment score was also used to enrich the significant GO terms of differentially expressed genes, where the top 3 processes were exogenous drug catabolic process, drug catabolic process and drug metabolic process in BP; membrane-bounded organelle, organelle and intracellular part in CC; steroid hydroxylase activity, monooxygenase activity, oxygen binding in MF (Figure 4). Furthermore, 64 and 26 KEGG pathways were identified in up and down regulated mRNAs (Supplementary Table S4). The top 10 KEGG pathways in dys-regulated mRNAs were shown in Figure 5, including phagosome, cell cycle and HTLV-infection at the top 3 of increased mRNAs while steroid hormone biosynthesis, linoleic acid metabolism and retinol metabolism at the top 3 of decreased mRNAs.

### qRT-PCR verification of selected mRNAs and circRNAs

**Figure 6:**
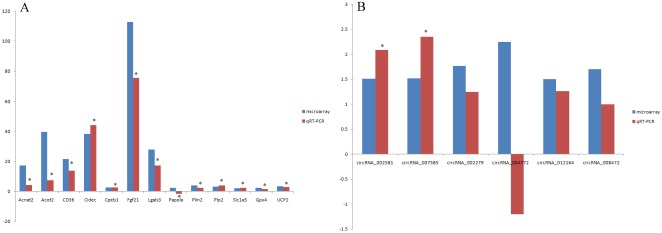
microarray and qRT-PCR analysis of selected mRNAs (A) and circRNAs (B) *, *p* < 0.01 in qRT-PCR verification when comparing data between NASH and control groups.

Of the 14 selected mRNAs (Acnat2, Acot2, CD36, Cidec, Cpeb1, Fgf21, Lgals3, Papola, Plin2, Plp2, Slc1a5, Gpx4, UCP2 and Gpnmb), only Papola had the opposite expression between microarray and qRT-PCR with statistic significance while the changes of the other 13 mRNAs were all verified by qRT-PCR (Supplementary Table S5). Besides, Gpnmb had the highest increased fold of 1095.02 in microarray and 686.15 in qRT-PCR (p<0.01). Since such up regulation level was 10 times higher than the other 13 mRNAs, we precluded it when visualizing the relative mRNA changes of microarray and qRT-PCR in Figure 6A. In contrast, the verification rate of circRNA by qRT-PCR was very low (Supplementary Table S6). Firstly, circRNA_008758 and circRNA_013447 were not able to be amplified after trying various primers, owing to the nature of short length. Secondly, of the left 6 circRNAs, only circRNA_004772 had the opposite expression in microarray and qRT-PCR but the difference is not statistically significant. Finally, though the data from microarray and qRT-PCR showed the same change tendency of circRNA_002581, circRNA_007585, circRNA_002279, circRNA_012164 and circRNA_008472, only the former two reached statistical significance and become candidates for further study (Figure 6B).

**Table 3:** Top 10 dys-regulated mRNAs in NASH

Gene name	Transcript	Fold change	*p* value
**Up regulation**			
Gpnmb	NM_053110	1095.019	3.87E-06
Fmo3	NM_008030	487.236	1.57E-05
Cyp4a14	NM_007822	444.738	1.06E-07
Ly6d	NM_010742	361.075	6.23E-05
Sult2a6	NM_001081325	350.101	1.12E-06
**Down regulation**			
Hsd3b5	NM_008295	5605.920	1.27E-07
Serpina4-ps1	NR_002861	3649.534	1.14E-06
Elovl3	NM_007703	426.055	2.64E-07
Susd4	NM_144796	237.364	5.18E-07
Pax2	NM_011037	123.988	2.05E-05

### circRNA-miRNA-mRNA construction

Based on previously verified qRT-PCR data, we predicted the downstream miRNAs of significantly increased circRNAs and the upstream miRNAs of significantly decreased mRNAs. Thereafter, those overlapped miRNAs were verified through qRT-PCR (Supplementary Table S7). Finally, four qRT-PCR verified circRNA- miRNA-mRNA pathways were constructed, including: circRNA_002581-miR-122- Slc1a5, circRNA_002581-miR-122-Plp2, circRNA_002581-miR-122-Cpeb1 and circRNA_007585-miR-326-UCP2. The effect of those regulation pathways in NASH needs further intensive study.

### Potential competition of circRNA with pre-mRNA

Since one genomic DNA may be able to alternatively transcribe into circRNA and linear mRNA and the transcription amount is complementary to each other, another function of circRNA may exist in competing with pre-mRNA. Therefore, we used Venny graph to make the intersection of tested mRNAs from mRNA microarray and predicted mRNAs as the linear alternative transcript of circRNAs. We found significantly increased Rn45s, Malat1 and zfp827 from mRNA microarray data while their associated circRNA_011775, circRNA_004300 and circRNA_001200 were significantly dereased from circRNA microarray. Similarly, Samd4, Wnk1, Enox1, Grb10 and Pkdcc were significantly decreased from mRNA microarray data while their associated circRNA_005305, circRNA_000390, circRNA_004772, circRNA_011381 and circRNA_016901 were significantly increased from circRNA microarray. Those circRNA-mRNA pair warrants further intensive investigation.

## DISCUSSION

Currently, NASH has been considered as a pivotal stage in NAFLD but its pathogenesis is still vague. Though firstly discovered in the cytoplasm of eukaryocyte and reported in 1979 by Hsu MT et al. [14], circRNA was neglected as the by-product from error alternative splicing in a long time. The importance of circRNA as molecular biomarker for disease diagnosis and as high efficient miRNA regulator has gradually been recognized. On one hand, the stable existence of circRNA in blood [15] and even saliva without blood constituent [16] have put weight on its application as non- invasive diagnostic marker; on the other hand, the landmark study by Hansen TB et al. [17] showed that ciRS-7 had over 70 miR-7 binding sites, supporting the role of circRNA as “miRNA sponge”. Since the circRNA profile and function of specific circRNA in NASH has never been reported, we combined the microarray screening, qRT-PCR verification and bioinformatics to fulfill this blank. The identified reservoir of circRNAs may provide preliminary data for NASH diagnosis as molecular biomarkers and the predicted circRNA-miRNA-mRNA network may shed light on NASH pathogenesis.

Since the function study of circRNA has just started in recent 5 years, it is not surprising that none of the top 10 dys-regulated circRNAs have been reported in the literature. However, some mRNAs that acted as the alternative transcription of those circRNAs were found to participate in liver diseases. For instance, Cam1 is the linear transcript of circ_002319 and previous report showed that its up regulation facilitated hepatocyte proliferation [18]. Pvt1 is the linear transcript of circ_011235 and was found to promote proliferation and stem cell property of liver cancer [19]. Besides, results from the intersection of circRNAs associated linear mRNAs and microarray identified mRNAs are much more encouraging. For instance, Malat1 was revealed to promote hepatic steatosis and insulin resistance by increasing nuclear SREBP-1c [20] while Samd4 was associated with uncoupled mitochondrial respiration, where mitochondrial dysfunction was the key factor in NASH pathogenesis [21].

Data from mRNA microarray were also informative. Of the top 10 dys-regulated mRNAs, Gpnmb had the highest fold change in the up regulated mRNAs and was previously identified as biomarker of NASH [22] while increased NLRP3 reinforced its role in NASH progression suggested by previous studies [23]. All these consistency reinforces the credibility of our results. Furthermore, Novel clues for NASH pathogenesis and progress were also identified. In increased mRNAs, UCP2 was found to participate in hepatic simple steatosis [24]; CIDEC was identified to promote alcoholic steatohepatitis in mice and humans [25] and Acot2 was located at mitochondria matrix and associated with lipid metabolism [26]. Results from GO process and KEGG pathway help to enrich and identify important mRNAs in NASH. In the top dys-regulated GO processes of decreased mRNAs, unsaturated fatty acid metabolic process was identified in BP while steroid hydroxylase activity, oxygen binding and oxidoreductase activity were found in MF. Concerning KEGG pathway, glycerolipid metabolism in increased mRNAs and inflammatory mediator regulation of TRP channels in decreased mRNAs showed their importance in NASH progression. All these findings provided novel clues for NASH study.

There are many works left to be done for circRNA study. For instance, though the mechanisms of circRNA formation were categorized into lariat-driven circularization, intron-pairing-driven circularization, circular intronic RNA and RBP or trans-factor driven circularization [27], which one takes major responsibility still needs investigation. Moreover, circRNA function research was complicated and further study is urgently needed, which generally includes following steps: firstly, determining specific circRNA by retrieving the circRNA full length with RACE amplification, gene sequencing, qRT-PCR, northern blot and fluorescence chromosomal in situ hybridization; secondly, determining the regulation of circRNA on miRNA by Myc-arg2 immunoprecipitation, RNA-pull down and dual luciferase reporter gene detection; finally, the phenotype change of specific disease after direct circRNA knockdown or over expression.

The circRNA-miRNA-mRNA network may serve as the powerful regulation pathway in NASH for the cascade amplification effect of circRNA-miRNA and miRNA-mRNA. However, in contrast to high qRT-PCR verification rate of mRNA (13 in 14), the successful qRT-PCR verification rate of circRNA microarray data was low (2 in 8), which may be due to the incomplete and short circRNA sequence. Though none of the verified 4 circRNA-miRNA-mRNA pathways have been reported previously, several molecules were involved in certain pathophysiologic processes when separately analyzed. For instance, miR-122 was a key factor and therapeutic target in liver disease[28] while miR-326 was validated as one of the independent prognostic predictor of hepatocellular carcinoma [29]. Concerning mRNAs, Slc1a5 was associated with hepatic glutamine uptake [30]; Plp2 was able to increase ER-stress induced neuronal apoptosis [31] while Cpeb1 was related with angiogenesis in chronic liver disease [32] and involved in induction of insulin resistance [33]. Lastly but most importantly, UCP2 participated in NASH through mitochondrial proton leak induction [34]. Nevertheless, none of the two circRNAs was previously reported. Therefore, research on these circRNA-miRNA-mRNA pathways would not only provide novel clues for NASH pathogenesis, diagnosis and treatment, but also establish a mature platform for further study in other diseases.

There are several shortcomings in this study that should be acknowledged. Firstly, the samples enrolled in this study was limited and we need a large number of subjects in an independent cohort study to verify our results and further decrease the scale of circRNA profile as diagnostic biomarker. Secondly, our results were based on animal level, whether it could be generalized into human beings is still questionable. Further study on the circRNAs of NASH patients and the comparison with our data is needed. Thirdly, we only studied the hepatic circRNA profile, while from the view of non-invasive diagnosis and the stable expression of circRNA in blood, it might be more meaningful to study the circulating circRNA of NASH patients. Finally, except for the effect of miRNA binding, circRNA was also able to facilitate gene transcription [35] and compete with pre-mRNA splicing [36], where those functions should be investigated in the future. To sum up, circRNA profile may serve as good candidate for NASH diagnosis and those identified circRNA-miRNA-mRNA pathways may provide novel mechanism and therapeutic targets for NASH.

## MATERIALS AND METHODS

### Ethic statement

This study was carried out in accordance with the recommendations in the Guide for the Care and Use of Laboratory Animals of the National Institutes of Health. The protocol on animal was approved by the institutional review board of the First Affiliated Hospital of Zhejiang University.

### NASH animal model construction

A total of 24 male BALB/c mice aged 6 week were commercially purchased (Cavens Lab Animal, Suzhou, china) and randomly divided into two groups: NASH (n=12) and control (n=12). All mice received food and water ad libitum and were maintained on a 12/12-h light/dark cycle. Control group was given a basic diet while NASH group was given a Methionine and choline deficiency (MCD) diet as previously reported [37]. Mice were sacrificed by neck dislocation at appointed time spot, where blood and liver tissue were collected for further analysis. After body weight detection, liver sections were stained with Haematoxylin-Eosin (H-E) and observed for hepatic steatosis and inflammation by Olympus microscope. Besides, histological activation index (HAI) was routinely calculated to semi-quantitatively evaluate the severity of hepatic injury [38]. Serum triglyceride (TG), alanine aminotransferase (ALT) and aspartate aminotransferase (AST) were tested with Hitachi 7600 clinical analyser in our hospital.

### RNA isolation and quality control

Total RNA was isolated from each liver tissue sample by separately mixing the sample with Polyacryl Carrier (MRC, OH, USA), TRIzol reagent (Invitrogen, Carlsbad, CA, USA) and chloroform, according to the manufacturer's protocol. RNA purification was routinely performed with an RNeasy Mini Kit (Qiagen, Hilden, Germany). RNA quantity was measured by a NanoDrop ND-1000 spectrophotometer (Thermo Fisher Scientific, MA, the USA) and RNA quality was tested with an Agilent 2100 Bioanalyzer (Agilent Technologies, Beijing, China).

### CircRNA and mRNA microarray analysis

Five samples from each group were randomly selected for microarray studies. Sample preparation and microarray hybridization were performed based on the Arraystar's standard protocols and each transcript was represented using 1-5 probes to improve the statistical confidence. For circRNA study, total RNA was digested with Rnase R (Epicentre, Madison, the USA) to remove linear RNA and enrich circular RNA. Thereafter, enriched circRNA was amplified and transcribed into fluorescent cRNA utilizing a random priming method with an Arraystar Super RNA Labeling Kit (Arraystar, Rockville, the USA). The labeled cRNA was hybridized onto Arraystar mouse circRNA Array V1.0. After slide wash, the arrays were scanned by the Agilent Scanner G2505C. For mRNA study, A whole mouse genome mRNA microarray (Agilent Technology, Santa Clara, CA, USA) was routinely carried out. Generally, sample labeling and array hybridization were performed according to the Agilent One-Color Microarray-Based Gene Expression Analysis protocol. Thereafter, Agilent Feature Extraction software (version 11.0.1.1) was used to analyze acquired array images. Quantile normalization and subsequent data processing were performed using the R software package. Differentially expressed circRNAs/mRNAs with statistical significance between two groups were identified through Volcano Plot and Fold Change filtering. Hierarchical Clustering was performed to show the distinguishable circRNA/mRNA expression pattern among samples.

### Computational analysis

Based on the significantly changed circRNA, the circRNA/miRNA interaction was predicted with Arraystar's home-made miRNA target prediction software based on previously established online analytical tool TargetScan [39] and miRanda [40], where the differentially expressed circRNAs within all the comparisons were annotated in detail with the circRNA/miRNA interaction information. For mRNA analysis, Gene Ontology (GO) that describes genes from any organism were used, covering the domains of Biological Process (BP), Cellular Component (CC) and Molecular Function (MF). Pathway analysis was carried out for a functional analysis of mapping genes to KEGG pathways. Fisher's exact/chi-squared test and FDR were used for significance detection, where p-value denotes the significance of GO term and pathway correlated to the conditions. The FDR indicates the false discovery rate; a smaller FDR indicates smaller error in judging the p-value. Finally, miRNAs as regulators of mRNAs were predicted by combination of TargetScan and miRanda.

### Quantitative real-time polymerase chain reaction (qRT-PCR) validation

Total isolated RNAs from NASH and control groups were reversely transcribed using a PrimeScript RT reagent Kit with gDNA Eraser (TaKaRa, Dalian, China). GAPDH was amplified as internal control and the relative amount of each circRNA/mRNA to GAPDH was calculated using the equation 2^−ΔCT^, where ^Δ^CT= C_TmiRNA_-C_Tu6_. In detail, based on combinational consideration of the fold change, raw data, FDR, p value and previously reported clinical manifestation, 14 mRNAs and 8 circRNAs were selected for qRT-PCR verification by a SYBR Green PCR kit (TaKaRa, Dalian, China) with 3 replication each. As shown in Supplementary Table S1, divergent primers (instead of commonly used convergent primers) were designed and optimized for circRNAs while primers for mRNAs were routinely applied. Moreover, the target specificity of PCR primer was verified using BLAST () and the appearance of a single-peak in the melting curve indicated the primer's specificity.

### CircRNA-miRNA-mRNA pathway construction and statistics

Logically, based on the binding capacity of circRNA on miRNA and miRNA on mRNA, the change of circRNA and mRNA should be in the same direction. Therefore, we chose those qRT-PCR verified increased circRNAs and mRNAs. Using Venny graph (http://bioinfogp.cnb.csic.es/tools/Venny/), the overlapped miRNAs predicted by circRNAs as downstream targets and by mRNAs as upstream regulators were enriched. Finally, the circRNA-miRNA-mRNA network was constructed. SPSS (version 16.0, Chicago, IL, USA) was used for statistical analyses. Data were expressed as the mean±standard deviation. Variables of the microarray and qRT-PCR data between the two groups were compared by Student's t-test. In the microarray results, a fold change of circRNA/mRNA ≥ 2.0 was chosen for further analysis and P<0.05 was considered as statistically significant.

## SUPPLEMENTARY MATERIAL TABLES














