# Supplementary material for: CircRNA expression pattern and circRNA-miRNA-mRNA network in the pathogenesis of nonalcoholic steatohepatitis
Source: Oncotarget. 2016 Sep 22;7(41):66455–67. doi: 10.18632/oncotarget.12186 (PMC5341813; doi:10.18632/oncotarget.12186)
Supplement: Supplementary file 1 [file oncotarget-07-66455-s001.doc]

CicrRNA expression pattern and cicRNA-miRNA-mRNA network in the pathogenesis of nonalcoholic steatohepatitis

^1^Xi Jin*****, ^2^Chun-yan Feng, ^2^Ruo-heng Zheng, ^1^Zun Xiang, ^1^Yi-peng Chen, ^1^You-ming Li

^1^Department of Gastroenterology, The First Affiliated Hospital, School of Medicine, Zhejiang University, Hangzhou, Zhejiang, China

^2^ Department of Internal Medicine, Zhejiang Medical College, Hangzhou, China

**Supplementary Table S1, qRT-PCR primer for circRNA and mRNA**

**1, qRT-PCR primer for circRNA**

| **gene** | **Sequence** | **Annealing**  **(℃)** | **Product length (bp)** |
| --- | --- | --- | --- |
| GAPDH(MOUSE) | F:5’ CACTGAGCAAGAGAGGCCCTAT3’  R:5’ GCAGCGAACTTTATTGATGGTATT3’ | 60 | 144 |
| mmu_circRNA_007585 | F:5’ CGAAATGCCCGAATGTCTG 3’  R:5’ CGGGATAATGAAATGAGGGAAG3’ | 60 | 187 |
| mmu_circRNA_002581 | F:5’ CCGCTCCGTGTTACACTCCC 3’  R:5’ TGACAGCGACACTGCTCTTCC 3’ | 60 | 148 |
| mmu_circRNA_012164 | F:5’ AAAGCCGTAAAGCCAAAGG 3’  R:5’ CGCGCTCTTGACCTTCTTG 3’ | 60 | 63 |
| mmu_circRNA_004772 | F:5’ CAGGACTGAACCGCACGAG 3’  R:5’ CAAGATCTCCCATCAGCAACC 3’ | 60 | 96 |
| mmu_circRNA_008472 | F:5’ AGAGAAGGAAAGACTTGGAGCAC3’  R:5’ GACACCAAAGCAGAGGACAAATA 3’ | 60 | 113 |
| mmu_circRNA_002279 | F:5’ TGGAAGTAATGTGGCAATGAGG3’  R:5’ AGACAGGAGGTTCCCTTGAGC3’ | 60 | 87 |
| mmu_circRNA_008758 | F:5’ AGAGGCTAGCAGTGGTCATTGTC3’  R:5’ TCGCGGGGCAACCTTTTCC3’ | 60 | 70 |
| mmu_circRNA_013447 | F:5’ TAAAGCCAAAGGCTGCCAAG 3’  R:5’ CGGCTTTAGACGCGCTCT 3’ | 60 | 51 |

**2,qRT-PCR primer for circRNA**

| **gene** | **sequence** | **Annealing**  **(℃)** | **Product length (bp)** |
| --- | --- | --- | --- |
| GAPDH(MOUSE) | F:5’ CACTGAGCAAGAGAGGCCCTAT3’  R:5’ GCAGCGAACTTTATTGATGGTATT3’ | 60 | 144 |
| Slc1a5 | F:5’ CCTGGTCTCCTGGATTATGTGG3’  R:5’ TATTTGCCGAGGCTGATGAAG3’ | 60 | 111 |
| Plp2 | F:5’ ATCACCTCCATTGTTGTCCTTGT3’  R:5’ TGCTGTATGTCTTTGCTGCTTTAG3’ | 60 | 138 |
| Cpeb1 | F:5’ CCATCCTGGACTCCCGTTCT3’  R:5’ CAGAGCTGAAGCCACTCGTGTC3’ | 60 | 60 |
| Papola | F:5’ GAAAGCAAGAATCTCCCACAATC3’  R:5’ CAACGCATCAATATCAGCACCT3’ | 60 | 111 |
| Gpnmb | F:5’ GATGCCAGAAGGAAGATGCTAAT3’  R:5’ CAGGTCAGATGTCAGTCCCAAAT3’ | 60 | 80 |
| Cidec | F:5’ AACCCTCAGGACTTTATTGGC3’  R:5’ CTTCATTGCAGCATCTTCAGAC3’ | 60 | 269 |
| Fgf21 | F:5’ GGAGGATGGAACAGTGGTAGGC3’  R:5’ AGGCTTTGACACCCAGGATTTG3’ | 60 | 104 |
| Acot2 | F:5’ TGGGAACACCATCTCCTACAAG3’  R:5’ CCACGACATCCAAGAGACCAT3’ | 60 | 98 |
| Acnat2 | F:5’ GCGGAGTGATTCTGTAGAGCAA3’  R:5’ TCACCAGCCTCGTTAGTCTTGTAG3’ | 60 | 246 |
| Lgals3 | F:5’ GAATGATGTTGCCTTCCACTTT3’  R:5’ ACTTGTATTTTGAATGGTTTGCC3’ | 60 | 150 |
| Gpx4 | F:5’ TGAGTGTGGTTTACGAATCCTG3’  R:5’ CTCCTTGATTTCTTGATTACTTCC3’ | 60 | 82 |
| Ucp2 | F:5’ TCCTGGAACGTAGTGATGTTTGTC 3’  R:5’ GGGAGGTGAGGTGGGAAGTAA 3’ | 60 | 208 |
| CD36 | F:5’ AAGCAAAGTTGCCATAATTGAGTC3’  R:5’ GGTCGATTTCAGATCCGAACA3’ | 60 | 191 |
| Plin2 | F:5’ TAGGCGTCTCTTTTCTCCAGG3’  R:5’ TGGGCAGGGCACTTGTCAT3’ | 60 | 231 |
